# An Explainable Multi-Modal Deep Learning Framework for Investigating the Influences of Dynamic Environmental Exposure on Momentary Stress

**DOI:** 10.1021/acs.est.6c09466

**Published:** 2026-09-21

**Authors:** Linsen Wang, Mei-Po Kwan, Liuyi Song

**Affiliations:** † Institute of Space and Earth Information Science, 26451 The Chinese University of Hong Kong, Shatin, Hong Kong Special Administrative Region 999077, China; ‡ Department of Geography and Resource Management, The Chinese University of Hong Kong, Shatin, Hong Kong Special Administrative Region 999077, China

**Keywords:** environmental exposure, momentary stress, spatiotemporal dynamics, multimodal deep learning, explainable AI

## Abstract

Urban stressors pose critical public health concerns by adversely affecting residents’ mental health. However, past studies have mostly focused on single-modal stressors under static exposure paradigms, with limited exploration of nonlinear environment-stress relationships. This study proposes an explainable multimodal deep learning framework that integrates remote sensing imagery (RSI), street-view imagery (SVI), mobile sensor data, and points of interest (POIs) to accurately predict and explain momentary stress in urban environments. Using 4342 momentary stress records and 125,440 min-level environmental exposure records collected in Hong Kong, we validated the framework, achieving an accuracy of 78.26% and an AUC-ROC of 82.77%. A multilevel gradient-based explainability analysis reveals that (i) environmental exposure within the most recent 25 min exerts the strongest influence on momentary stress and notable spatial heterogeneity exists in stress prediction, highlighting the importance of capturing exposure dynamics; (ii) specific environmental thresholds were identified, including 55 dBA for noise, 25 μg/m^3^ for PM_2.5_, 32 °C for temperature, 40%–70% for humidity, and 25% for greenery visibility; and (iii) from an aerial perspective, open spaces consistently suppress stress, while the influence of buildings varies depending on surrounding spatial configuration. This study provides an integrative and explainable tool offering actionable insights for public health interventions.

## Graphical abstract

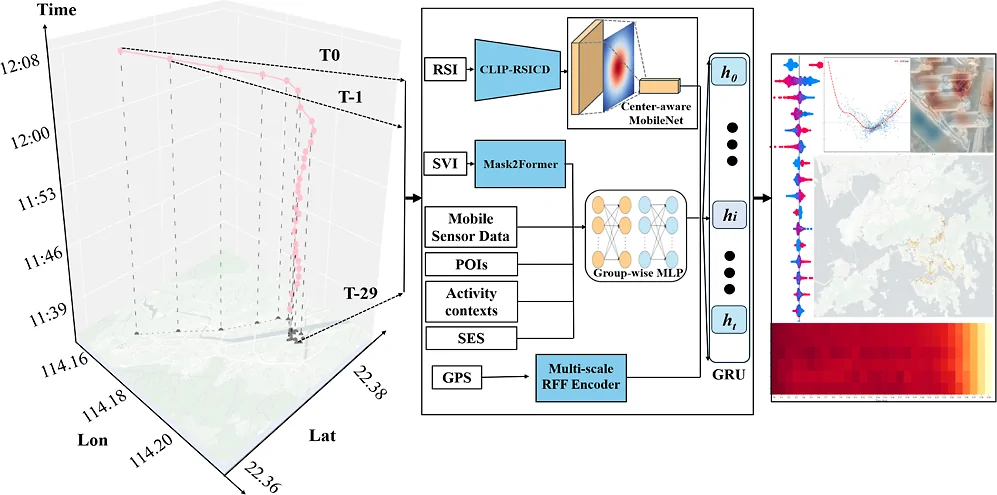

## 1 Introduction

Urban stress has emerged as a critical public health concern, with various environmental exposures being stressors for urban residents. These exposures encompass greenery deprivation, noise pollution, air pollution, heatwaves, and high-density built environment, all of which can trigger stress response.^1–6^ These stressors can provoke psychological and physiological stress responses, such as hypothalamic-pituitary-adrenal axis activation, autonomic nervous system dysregulation, and chronic low-grade inflammation, which are associated with various detrimental health outcomes, including mental health disorders, cardiovascular disease, metabolic dysfunction, and cognitive decline.^7–9^

The field of urban stress research is experiencing a paradigm shift. Traditionally, the focus has mostly been on chronic stress and its association with static environmental characteristics. The conventional static research paradigm faces methodological challenges of the uncertain geographic context problem (UGCoP) and the neighborhood effect averaging problem (NEAP).^10,11^ Static measures fail to capture the spatiotemporal variability of an individual’s daily mobility, ignoring the fact that environmental exposure is not constant but fluctuates dynamically as individuals move through diverse urban spaces.^12–14^ However, there is growing recognition of the importance of momentary mental wellbeing. It refers to a person’s psychological state as experienced at the moment and in context, encompassing momentary affect, mood, and stress, and typically assessed through brief self-reported momentary ratings.^15–17^ Momentary stress is a commonly assessed aspect of momentary mental wellbeing and is closely linked to environmental stressors.^18–20^ It has been widely operationalized using brief self-report measures, allowing temporally immediate and context-specific assessment that is sensitive to dynamic changes in real-world daily life. This matters for urban stress research because stressors are transient, varying from minute to minute as residents move through the city. Temporal immediacy, situational specificity, and dynamic responsiveness therefore become essential properties of the measure, allowing momentary assessments to reveal exposure-response relationships that chronic or retrospective measures average out.^15^

Ecological momentary assessment (EMA) has emerged as the gold-standard methodology for capturing momentary stress, enabling us to link real-time environmental exposures with instantaneous stress responses.^15,21,22^ Such an approach facilitates more nuanced analysis of the complex environment-stress relationship. Recent studies have leveraged massive spatiotemporal data (e.g., remote sensing imagery [RSI], street-view imagery [SVI], points of interest [POIs], and GPS-enabled mobile sensor data) to extract urban stressors and stress-relief elements, pairing these with EMA-measured momentary stress to reconstruct urban environment-stress scenarios.^19,23–26^ Deep learning has further been applied to fuse on-body, environmental, and location sensor streams for inferring emotional states.^16,27,28^ However, existing research predominantly focuses on single-modal approaches (e.g., greenery derived from RSI or SVI, noise or air pollution captured by mobile sensors) with limited integration of multimodal urban environmental data in momentary stress studies. This gap hinders our ability to accurately model the relationship between urban environments and momentary stress. Different urban modalities offer complementary perspectives on the urban environment. For instance, SVI provides fine-grained information about eye-level visual stressors but lacks consideration of spatial morphology. RSI enhances eye-level views by providing consistent aerial perspectives for analyzing spatial relationships and landscape patterns.^29^ Similarly, POIs and activity diaries can provide essential contextual information about urban functions and activities where momentary stress is present. For example, high noise levels in residential areas may constitute a stressor, whereas the same noise levels in restaurants might have neutral or even negative associations with stress. Further, although various statistical models (e.g., linear models, generalized linear models [GLMs]) have been employed to examine urban environment-momentary stress relationship,^19–21,23–26,29^ the complex nonlinear response curves and time-lag effects characterizing environment-stress dynamics remain insufficiently explored. Traditional linear approaches may fail to capture threshold effects and interaction effects among multiple environmental stressors, and the lagged physiological and psychological responses to environmental exposures.^30^

To address these limitations, this study proposes an innovative multisource fusion framework that integrates SVI, RSI, POIs, and mobile sensor data, employing deep learning techniques to model and explain residents’ momentary stress. Moving beyond single-modal and linear analyses, the framework provides a methodological foundation for examining the nonlinear, time-lagged, spatially heterogeneous, and morphological associations between urban environmental exposures and momentary stress. We collected 2 days of EMA momentary stress data from 800 participants in Hong Kong, yielding 4342 momentary stress records, along with GPS trajectories, noise levels, PM_2.5_ concentrations, temperature, humidity measurements, and activity contexts from the preceding 30 min, totaling 125,440 min-level records. Corresponding to these GPS locations, we obtained 19,420 remote sensing and street-view images, as well as POI compositions around geographic coordinates. Leveraging pretrained state-of-the-art models to extract image embeddings and integrating deep learning architectures, we developed a multimodal fusion model that enables both the prediction of momentary stress and the explanation of nonlinear relationships between environmental exposures and stress responses.

## 2 Materials and Methods

### 2.1 Data

#### 2.1.1 Mobile Survey Data

Mobility-based data from 800 participants was collected between November 2021 and March 2023. Participants live in four areas: Sha Tin, Central and Western, Kwai Tsing, and Kwun Tong. Each area consists of several census tracts in Hong Kong. These four areas were selected to encompass the extensive environmental diversity of Hong Kong (see Text S1). Participants were selected through a stratified sampling method informed by the local population census (Table S1), and all provided written informed consent. The recruitment and survey procedures are described in detail in Text S2. Ethical approval was obtained from the Survey and Behavioral Research Ethics Committee at the authors’ university (Protocols SBRE-19-123 and SBRE­(R)-21-005). Each participant was equipped with a device set for a 48 h monitoring period spanning 1 weekday and 1 weekend day: a GPS-enabled smartphone (Handy, Android), a portable noise meter (SLM-25) recording the A-weighted equivalent continuous sound level (LAeq, dBA), and an ambient sensor (AirBeam) monitoring PM_2.5_ (μg/m^3^), temperature (°F), and humidity (%). Full instrument standards are listed in Text S2. This 48 h duration design balanced coverage of typical weekday and weekend contexts with the burden of continuous multisensor monitoring. Similar 48 h designs have been used in mobility-based environmental health studies.^21,25,31,32^ Moreover, participants received ecological momentary assessment (EMA) prompts at three fixed time points daily (12:00, 16:00, 20:00) to report momentary stress levels, and completed an activity-travel diary each evening. These time points were selected to span midday, late afternoon, and evening while providing comparable assessments across participants and limiting interruptions. Momentary stress was assessed with a single item, “Do you feel stressed right now?”, rated on a 6-point scale from 1 (“not at all”) to 6 (“very severe”). These prompts were signal-contingent, delivered at fixed times rather than triggered by specific events, capturing momentary stress at the sampled times rather than emergent responses to discrete stressor events. Details of the stress measure, the activity-travel diary, and assessment completeness are provided in Text S2. Since this study focuses on momentary stress and immediate environmental exposure, we extracted GPS coordinates, LAeq, PM_2.5_, temperature, and humidity from the 30-min window preceding each EMA record and integrated them into minute-level sequences. For the predictive analysis, the 6-point stress responses were recoded into a binary outcome (no stress vs stress), as respondents have difficulty distinguishing intermediate and ambiguous scores. Subjective responses to the urban environment are complex judgments that are typically captured through coarse perceptual constructs rather than fine continuous scores.^33^ A sensitivity analysis also confirmed the binary formulation to be the most reliable representation (Table S3). The resampling, aggregation, and cleaning procedures, together with the associated sensitivity analyses, are detailed in Text S3. The data structure of the cleaned and integrated data set remained largely consistent with that of the raw data (Tables S1 and S2).

#### 2.1.2 Remote Sensing Imagery, Street-View Imagery, and Points of Interest

The cleaned GPS coordinates (Section 2.1.1) yielded 19,420 unique coordinate pairs, which served as the spatial basis for acquiring RSI, SVI, and POI. High-resolution remote sensing imagery was obtained from Mapbox’s global raster imagery repository, a composite data set that integrates aerial products from a range of providers, including the National Aeronautics and Space Administration (NASA), the United States Geological Survey (USGS), Maxar, and Nearmap. Following a conventional approach,^34^ for each location, we retrieved image tiles at zoom level 17 to capture the surrounding urban context. Each tile covers a 600 m × 600 m ground area, with a spatial resolution of 0.6 m and a size of 1024 × 1024 pixels. Eye-level SVI was obtained from Google Maps as 640 × 640-pixel images captured at four azimuth angles per location and stitched into a composite panoramic view. POIs were obtained from Amap, a widely used digital mapping platform in Hong Kong, and grouped into eight functional categories based on the city’s spatial functional classifications (Table S6). For each location, we computed the POI composition and the total number of POIs within a 300 m buffer.^25^ Further acquisition details are provided in Text S4.

### 2.2 Multimodal Deep Learning Modeling Framework

This study proposes a multimodal deep learning approach for modeling momentary stress in urban environments, aiming to achieve accurate prediction and interpretation by integrating complementary mobile sensor data, multiview imagery, and pertinent contextual features (see the GitHub repository for specific code: https://github.com/GEO-AI-for-health/Multi-Modal-Deep-Learning-Framework).^35^ Figure 1 provides an overview of the framework. The aerial RSI captures the surrounding urban form and land cover, such as the spatial configuration of built-up, green, and open space. The RSI is encoded by a CLIP-based vision encoder,^36^ a pretrained image model that converts each image into a numerical feature vector, with a center-aware spatial adapter^37^ that emphasizes the participant’s immediate surroundings. This vector encodes the morphological characteristics of the surrounding built environment that inform the prediction. The eye-level SVI captures the streetscape a person actually sees, including the visible proportions of greenery, sky, buildings, and traffic that prior work links to psychological restoration or stress.^19,25^ The SVI is processed by semantic segmentation,^38^ which labels every pixel by object type such as road, building, or vegetation. The mobile-sensor readings (noise, PM_2.5_, temperature, and humidity) capture in situ physical exposures with established links to discomfort and stress,^23–26^ whereas the POI composition, activity context, and socioeconomic status (SES) capture the functional, behavioral, and personal setting in which exposure occurs. These structured features are processed by a group-wise multilayer perceptron (MLP), a standard feed-forward network applied to each feature group. The GPS coordinates are encoded by a multiscale random Fourier feature encoder,^39–41^ which represents coordinates as smooth spatial features at several scales. Because exposure varies over short time scales, the encoded modalities are computed at each of the 30 1-min steps preceding a stress report, concatenated, and modeled sequentially by a two-layer gated recurrent unit (GRU),^42^ a recurrent network that captures how recent exposure history relates to momentary stress. Full architectural details, including layer dimensions, are provided in Text S5.

**Figure 1:**
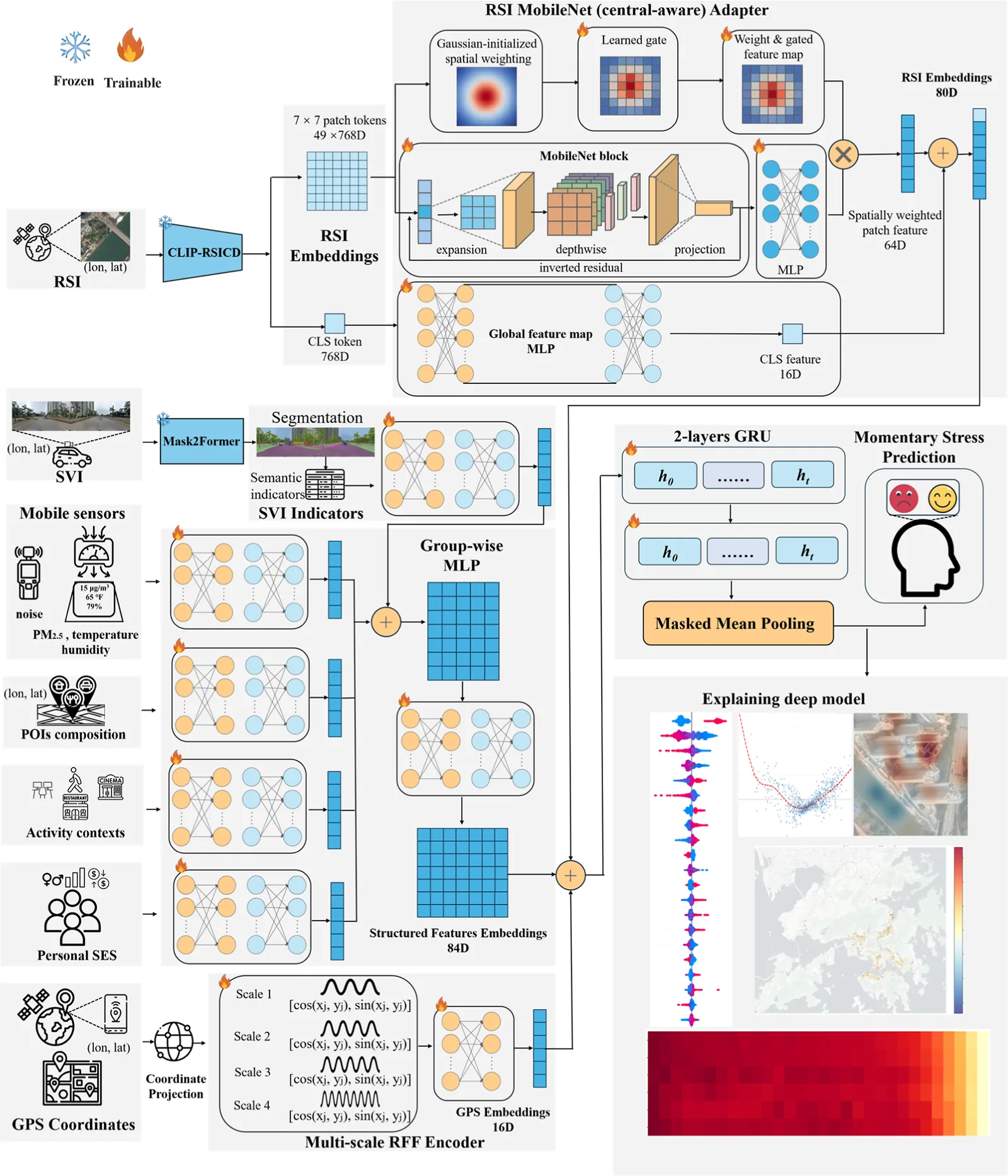
Overview of the proposed explainable multimodal deep learning framework. Seven input modalities (RSI, SVI, mobile-sensor readings, POI composition, activity context, socioeconomic status, and GPS coordinates) are each encoded into numerical representations, concatenated at every 1-min step, and modeled across the 30-min window preceding each stress report by a two-layer GRU to predict momentary stress. A SHAP (SHapley Additive exPlanations)-based explainability module (right) attributes each prediction to features, time steps, locations, and image regions.

For RSI, we used the CLIP-RSICD-v2 vision encoder to convert each satellite image into a numerical embedding, as shown in eq 1

1 $$E_{RSI}=f_{CLIP‐RSICD}\left(Resize\left(I_{RSI}\right)\right)\in R^{50\times 768}$$

where *I*_RSI_ denotes the input remote sensing image and *E*_RSI_ represents the resulting embedding, comprising one CLS token and 49 patch tokens (7 × 7 spatial grid). This step converts each satellite image into a fixed-length numerical representation of its visual content, which the center-aware spatial adapter then compresses into the 80-dimensional RSI embedding used downstream (Text S5).

For SVI, we used Mask2Former to assign a semantic label to every pixel of the panoramic image, as formulated in eq 2

2 $$M_{SVI}=f_{Mask2Former}\left(I_{SVI}\right)\in {\left\{0,1,...,18\right\}}^{H\times W}$$

where *I*_SVI_ denotes the input panoramic SVI and *M*_SVI_ is the predicted semantic label map. The pixel-level predictions were then aggregated into category-wise proportions, as defined in eq 3

3 $$x_{SVI}={\left[\frac{n_{c}}{H\times W}\right]}_{c\in C}$$

where *n*_*c*_ = |{(*i*,*j*):*M*_SVI_(*i*,*j*) = *c*}|denotes the pixel count for category *c*, and *C* = {road, sidewalk, building, sky, greenery, person, rider, vehicle}.

The SVI indicators, the mobile-sensor readings, the POI compositions, the activity context, and SES were organized into five semantically coherent feature groups and processed by a group-wise MLP. The GPS coordinates were encoded by the multiscale random Fourier feature encoder. At each 1-min step, the RSI, structured, and GPS embeddings were concatenated and processed by the two-layer GRU, followed by masked mean pooling to handle variable-length sequences and a linear classification head. Because the stress labels were class-imbalanced, the model was trained with a weighted focal loss, as defined in eq 4

4 $$L=-\frac{1}{N}\sum_{i=1}^{N}\alpha_{y_{i}}{\left(1-p_{t,i}\right)}^{\gamma }\log\left(p_{t,i}\right)$$

where *α*_*yi*_ is the class-balancing weight, *p*_*t*,*i*_ is the predicted probability for the ground-truth class, and γ is the focusing parameter. The model was optimized with Adam with decoupled weight decay.^43^

To systematically evaluate the contribution of each architectural component and input modality, we conducted two ablation studies, varying the spatial adapter and the temporal encoder and removing one input modality at a time. Model performance was evaluated by 10-fold cross-validation using accuracy, precision, recall, *F*1-score, and area under the receiver operating characteristic curve (AUC-ROC). The compared configurations and the cross-validation setup are detailed in Text S5.

### 2.3 Model SHAP-Based Explainability Analysis

To provide explainable insights into the environmental determinants of momentary stress, we employed gradient explainer.^44^ It is a gradient-based SHAP (SHapley Additive exPlanations) method that computes expected gradients by averaging over background baselines sampled from the training data. For a given input *x* and background distribution D , the SHAP attribution for each feature *j* is defined in eq 5

5 $$\phi_{j}\left(x\right)=E_{x^{\prime }∼D}\left[\left(x_{j}-x_{j}^{\prime }\right)\cdot \frac{\partial f\left(\mathrm{x̃}\right)}{\partial {\mathrm{x̃}}_{j}}{|}_{\mathrm{x̃}=x^{\prime }+\alpha \left(x-x^{\prime }\right)}\right],,\alpha ∼U\left(0,1\right)$$

where *f*(·) denotes the stress-class logit. Positive values indicate stress-promoting contributions, while negative values indicate stress-suppressing effects.

Building on these attributions, we conducted four levels of explainable analysis (Figure 1). First, at the feature level, we examined the structured inputs at the time step closest to the EMA report (*T* = 0). Features were ranked by their mean absolute SHAP value to indicate overall importance. Dependence plots, fitted with a generalized additive model (GAM), were used to reveal nonlinear, threshold-type relationships between a feature’s value and its SHAP contribution. Second, at the modal-temporal level, we examined how the importance of each modality evolves across the 30 1-min steps, visualized as a temporal heatmap. Third, at the spatial level, we mapped the SHAP attribution of each GPS location geographically, distinguishing stress-promoting from stress-suppressing places. Fourth, at the image level, we attributed predictions to specific regions of the satellite imagery on the 7 × 7 patch grid and overlaid these attributions on the original images. Further details, including the background-sampling procedure, are provided in Text S6.

## 3 Results

### 3.1 Model Performance and Ablation Experiments

To validate the effectiveness of the model architectural components, we compared combinations of representative spatial adapter types (NoAdapter, Attention, CNN, MobileNet without center-aware weighting, and center-aware MobileNet) and temporal encoders (GRU, LSTM, and MLP). Model performance was evaluated using accuracy, precision, recall, *F*1-score, and AUC-ROC. Table 1 presents the mean performance metrics across 10-fold cross-validation for different spatial adapter and temporal encoder configurations. The results indicate that MobileNet is the optimal spatial adapter for RSI feature compression, and GRU is the most effective temporal encoder for capturing temporal dependencies. Further, incorporating the center-aware mechanism into MobileNet yielded an improvement of approximately 2% in accuracy, 7% in precision, and 2.5% in AUC-ROC, despite modest declines in recall and *F*1-score. The confusion matrix and ROC curve further corroborate these findings (Figures S1 and S2).

**Table 1:** Model Comparison by Varying Spatial Adapters and Temporal Encoders

	metric				
model	accuracy	precision	recall	*F*1-score	ROC-AUC
NoAdapter-GRU	0.6856	0.7239	0.7066	0.7152	0.7316
Attention-GRU	0.6355	0.6813	0.6527	0.6667	0.6473
CNN-GRU	0.6856	0.7239	0.7066	0.7152	0.7316
MobileNet-MLP	0.7291	0.7945	0.6946	0.7412	0.8249
MobileNet-LSTM	0.7492	0.7987	0.7365	0.7664	0.8242
MobileNet (w/o center-aware)-GRU	0.7625	0.7474	0.8683	0.8033	0.8133
MobileNet-GRU	0.7826	0.8148	0.7904	0.8024	0.8277

To assess the effectiveness of multimodal features in predicting momentary stress, we conducted a modality ablation study using the optimal model (center-aware MobileNet-GRU), in which we began with the full model using all seven modalities and then removed one modality at a time to quantify the marginal contribution of each. As shown in Table 2, the modality ablation study reveals that RSI embeddings are the strongest individual predictors. The deep model relies heavily on these embeddings; removing the RSI modality leads to substantial performance degradation across all metrics, with precision declining by approximately 10% and accuracy by 8.7%. This highlights the powerful advantage of RSI in capturing the surrounding urban morphology (e.g., building density, landscape patterns) from an aerial perspective for momentary stress prediction. GPS embeddings emerge as the second strongest predictors. Removing this modality results in a 5.9% loss in precision and a 5.4% loss in accuracy, suggesting that geographic location exerts a considerable influence on momentary stress and implying notable spatial heterogeneity in stress experiences across urban areas.

**Table 2:** Modality Ablation Model Performance

	metric				
modality	accuracy	precision	recall	*F*1-score	ROC-AUC
all	0.7826	0.8148	0.7904	0.8024	0.8277
(w/o) GPS	0.7291	0.7560	0.7605	0.7582	0.8053
(w/o) RSI	0.6957	0.7159	0.7545	0.7347	0.7544
(w/o) SVI	0.7559	0.7733	0.7964	0.7847	0.8147
(w/o) mobile sensors	0.7759	0.7874	0.8204	0.8035	0.8284
(w/o) POIs	0.7458	0.7078	0.9281	0.8031	0.7902
(w/o) activity contexts	0.7425	0.7922	0.7305	0.7601	0.8115
(w/o) personal SES	0.7324	0.7843	0.7186	0.7500	0.8165

Activity contexts and personal SES rank as the third and fourth most influential modalities, respectively, indicating that the type of activity being undertaken and individual-level characteristics are also non-negligible predictors. SVI indicators and POI composition follow in importance. Their removal also leads to measurable performance decline, demonstrating that eye-level streetscape features and urban functional contexts serve as valuable complements for accurate momentary stress prediction. Mobile sensor data exhibits the smallest impact on model performance upon removal. This may be because the environmental conditions captured by these sensors (e.g., noise and air quality) are partially encoded within the RSI, SVI, and POI modalities. Nevertheless, mobile sensor data contributes to a non-negligible momentary stress prediction improvement, particularly in precision. These observations are further supported by the confusion matrix and ROC curve analyses (Figures S3 and S4).

### 3.2 Spatiotemporal Patterns of Environmental Exposure Effects on Momentary Stress

To investigate the time-lagged effects of environmental exposure on momentary stress prediction, we computed input-gradient attributions for GPS and RSI modalities and applied Gradient Explainer for structured feature groups based on the optimal MobileNet-GRU model. The resulting importance profiles across the 30-min window were visualized as a temporal heatmap (Figure 2a). The results reveal that all modalities exhibit relatively consistent temporal patterns: environmental exposure at the most recent time step (T0) exerts the strongest influence on momentary stress prediction, with the effect diminishing progressively further back in time. The temporal heatmap displays a distinct inflection pattern, with the specific transition point varying slightly across modalities. Overall, environmental exposure within the most recent 25-min window (T-24 to T0) contributes substantially to momentary stress prediction, whereas exposure beyond 25 min (T-25 to T-29) shows comparatively weaker influence. Notably, the inflection points for activity contexts and SVI indicators occur earlier (around T-26), suggesting a stronger temporal dependency in the influence of these features. In contrast, mobile sensor data exhibits a more proximal inflection point (around T-21), implying a more immediate effect on momentary stress prediction. This may be attributable to the greater temporal volatility inherent in these sensor measurements.

**Figure 2:**
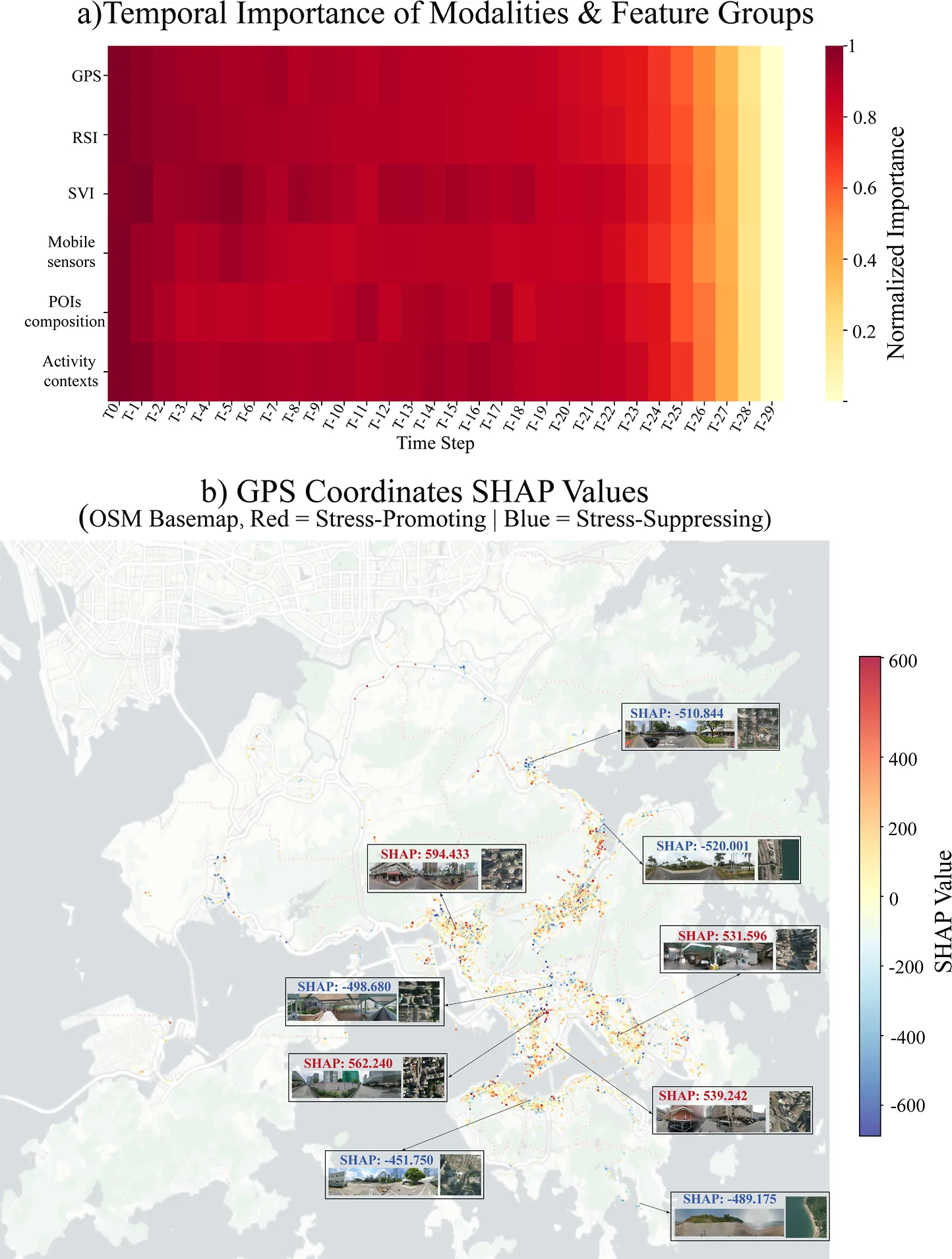
Spatiotemporal patterns of environmental exposure effects on momentary stress. (a) Temporal importance of the input modalities and feature groups, showing the normalized SHAP-based importance of each modality at each of the 30 1-minute steps preceding the stress report (T0, most recent, to T-29, most distant). (b) Location-level SHAP values for the GPS coordinates, where red denotes stress-promoting locations (positive SHAP) and blue denotes stress-suppressing locations (negative SHAP). The RSI and SVI of eight representative locations, spanning both stress-promoting and stress-suppressing values, are shown together with their location-level SHAP values.

To explore the spatial patterns of momentary stress based on the deep learning model, we conducted GPS SHAP-based spatial analysis, deriving location-level attributions and mapping them geographically to distinguish between stress-promoting and stress-suppressing areas. Figure 2b illustrates the spatially varying influence of geographic coordinates on momentary stress prediction, where blue indicates locations that reduce the predicted probability of stress and red indicates the opposite. The results reveal pronounced spatial heterogeneity in momentary stress, with geographically proximate locations exhibiting markedly different, and even opposing, influences on stress prediction. This may be related to Hong Kong’s highly mixed land use patterns, elevated building density, and high plot ratios, which give rise to considerable heterogeneity in the spatial context of the urban landscape. Further, the results indicate that areas connecting suburban districts and urban clusters tend to be associated with lower predicted stress probabilities, whereas central urban areas exhibit the opposite pattern.

### 3.3 Feature Importance, Nonlinear Relationships, and Spatial Attribution in Momentary Stress Prediction

To explain the importance of and nonlinear relationships between structured features and momentary stress prediction within the deep learning model, we applied feature-level SHAP analysis at the time step closest to the EMA record (*T* = 0), as the temporal importance analysis (Section 3.2) identified this step as the most influential. This yielded global importance rankings based on mean absolute SHAP values, alongside dependence plots fitted with GAMto characterize the nonlinear associations between individual features and stress prediction. As shown in Figure 3a, the results indicate that activity contexts and personal SES are prominent among the top-ranked features (i.e., the SHAP values span the widest ranges): work/study is the most important predictor, followed by age, with family income and education level also ranking in the top five. Notably, restaurant POIs rank third overall, highlighting the relevance of surrounding dining-related urban functions to momentary stress prediction. Among the SVI indicators, greenery ranks highest (sixth overall), suggesting that visible green space is the most salient streetscape feature for stress prediction. Mobile sensor readings, such as noise and temperature, also appear in the upper tier, whereas SVI indicators related to the built infrastructure (e.g., building, sidewalk, sky) and POI categories, such as industry, recreation/tourism, and total POI count, rank near the bottom, indicating relatively limited marginal contributions.

**Figure 3:**
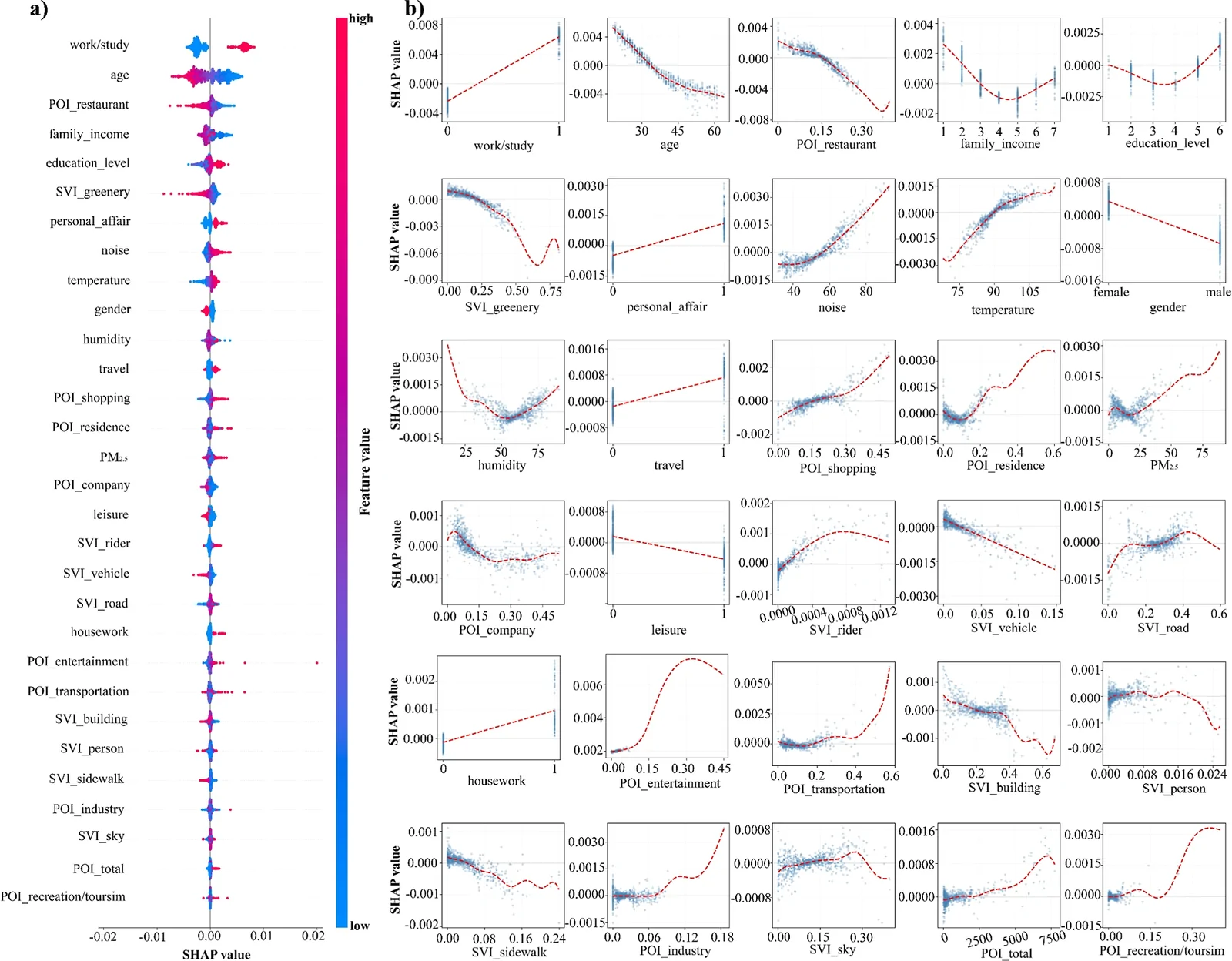
Feature-level explainability analysis of the structured features (SVI indicators, mobile-sensor readings, POI composition, activity context, and personal SES), computed at the time step closest to the stress report (T0). (a) Global feature importance, with features ranked by their mean absolute SHAP value. (b) SHAP dependence plots showing how each feature’s value relates to its SHAP contribution; red dashed curves denote GAM-fitted trends. Family income was measured on a 7-level ordinal scale (1 = less than HKD 9999; 2 = 10,000–19,999; 3 = 20,000–29,999; 4 = 30,000–39,999; 5 = 40,000–59,999; 6 = 60,000–79,999; 7 = over 80,000 per month). Education was measured on a 6-level ordinal scale (1 = primary or below; 2 = secondary; 3 = postsecondary diploma/certificate; 4 = postsecondary subdegree; 5 = postsecondary degree; 6 = master’s or above).

Figure 3b displays the SHAP dependence plots for all 30 structured features. Among activity contexts, work/study exhibits the strongest association, with engagement in work or study activities substantially increasing the predicted probability of stress. Personal affairs and housework also show a positive association, with engagement in personal affair or housework elevating stress prediction. Travel displays a modest positive trend, suggesting that commuting may slightly elevate stress levels. Leisure shows a weak negative association, indicating a marginal stress-buffering effect. For personal SES, age demonstrates a clear negative nonlinear trend, with younger participants associated with higher predicted stress and the effect gradually attenuating with increasing age. Both family income and education level display nonlinear U-shaped patterns, where moderate levels are associated with the lowest predicted stress, while both lower and higher ends correspond to elevated stress predictions. Females are associated with slightly higher stress predictions than males.

For mobile sensor readings (ambient environment), noise shows a positive trend with a notable threshold around 55 dBA, beyond which the predicted probability of stress increases markedly. Temperature displays a weak U-shaped association, though the left branch (lower temperatures) is less pronounced due to the limited availability of low-temperature observations in Hong Kong’s subtropical climate. The effect becomes more evident above approximately 90 °F (32.22 °C), where higher temperatures are associated with a sharper increase in stress prediction. Humidity demonstrates a more pronounced U-shaped pattern, with thresholds at approximately 40% and 70%. Humidity levels below 40% or above 70% are both associated with elevated stress predictions, while moderate humidity corresponds to the lowest stress. PM_2.5_ shows a largely monotonic positive association, with a discernible threshold around 25 μg/m^3^, above which the predicted probability of stress increases substantially.

For SVI indicators, greenery exhibits a clear negative relationship, with a notable threshold at approximately 25%. Once the proportion of visible greenery in the streetscape exceeds this level, the predicted probability of stress decreases substantially. Vehicles show a negative linear trend, where greater vehicle presence in the streetscape is associated with lower predicted stress. Roads display a weak positive association. Rider shows a weak inverted-U pattern. Buildings, persons, sidewalks, and sky display relatively flat curves with limited effects. For POI composition, restaurant ranks notably high with a negative nonlinear trend, where a higher proportion of restaurant POIs in the surrounding area is associated with reduced stress prediction, suggesting a potential stress-buffering effect of dining-related urban amenities. Shopping-related POIs exhibit a positive association, with a higher proportion of shopping-related POIs corresponding to increased stress prediction. Residence-related POIs display a weak U-shaped pattern, with both lower and higher proportions slightly elevating stress. Company-related POIs show a negative association, where a higher proportion of company-related POIs is linked to reduced stress prediction. Entertainment and transportation POIs both exhibit positive associations with discernible thresholds at approximately 15% and 40%, respectively, beyond which the predicted probability of stress increases notably. Industry, total POI count, recreation/tourism POIs, and the remaining categories display relatively flat dependence curves with limited nonlinear effects.

To explain the spatial attribution of surrounding urban features from an aerial perspective on momentary stress prediction, we computed the mean SHAP value across all dimensions for each spatial patch of the RSI embeddings and overlaid the resulting attribution maps onto the original RSI. Figure 4 presents 12 representative patch-level RSI SHAP attribution maps. Several consistent spatial patterns emerge across the selected images. Vegetation and green spaces are predominantly associated with blue attributions. Tree canopies, vegetated hillsides, and park areas consistently exhibit stress-suppressing effects, corroborating the strong negative relationship between greenery and stress identified in the feature-level analysis. Open spaces and recreational facilities, such as sports grounds and waterfront areas, are also highlighted in blue, consistent with the restorative environment hypothesis. Road infrastructure and transportation nodes exhibit context-dependent attributions. Major multilane roads and highway interchanges tend to show red attributions, likely reflecting traffic-related stressors. However, quieter road segments adjacent to vegetation may show neutral or blue attributions, suggesting that the surrounding environmental context modulates the stress influence of transportation infrastructure. Buildings display heterogeneous attribution patterns that appear to be shaped by urban morphological context rather than mere presence. Densely packed high-rise clusters and buildings situated near major road intersections tend to exhibit red attributions, whereas lower-density buildings surrounded by greenery or open space are more frequently associated with blue or neutral attributions. This suggests that the model captures the broader morphological context, including building density, spatial arrangement, and proximity to green or open spaces, rather than simply responding to the presence of buildings.

**Figure 4:**
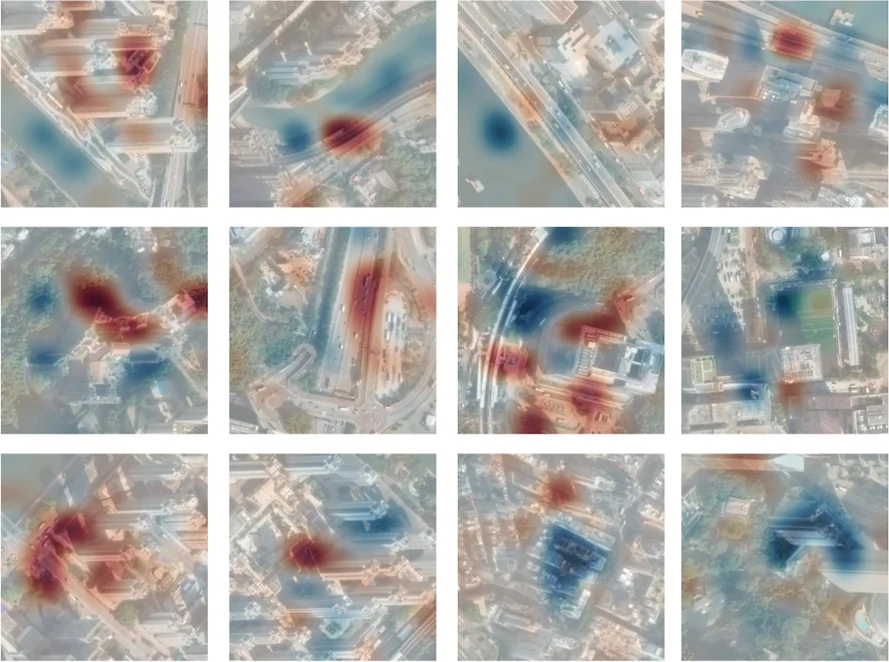
Patch-level SHAP attribution maps for the RSI, overlaid on the original images. Attributions were computed for the 49 patch tokens (7 × 7 grid) and upsampled onto the image. Red patches indicate regions that pushed the prediction toward stress (positive SHAP), and blue patches indicate regions that pushed it toward no stress (negative SHAP).

## 4 Discussion

This study proposed a multimodal deep learning framework that innovatively integrates SVI, RSI, POIs, and mobile sensor data to model and explain urban residents’ momentary stress. Using mobile survey data collected from 800 participants in Hong Kong and corresponding massive spatial data, we validated the framework’s reliability, with the optimal model configuration achieving an accuracy of 78.26% and an AUC-ROC of 82.77%. The modality ablation study demonstrates that the full multimodal model achieves the highest accuracy, confirming that the proposed framework successfully fuses complementary perspectives from multiple data sources and overcomes the limitations inherent in single-modal approaches. To our knowledge, this is the first study to simultaneously fuse multiperspective imagery (aerial and eye-level), mobile sensor data, and urban functional contextual features within a unified deep learning architecture for momentary stress prediction and explanation, representing a methodological complement to prior single-modal or statistical approaches.^19,20,24,25^ Rather than serving as a causal identification framework, this approach complements experimental and observational designs by enabling the characterization of nonlinear, time-lagged, spatially heterogeneous, and urban morphological patterns in exposure-stress relationships under real-world mobility conditions.

A key innovation of this framework lies in its ability to move beyond conventional feature-engineered representations of the urban environment. Unlike previous studies that relied on manually extracted indicators from RSI or SVI,^25,26,29^ our approach leverages pretrained vision encoders to extract high-dimensional embeddings that preserve rich spatial and semantic information. The modality ablation study confirms that RSI embeddings constitute the strongest predictor, highlighting the critical role of aerial-perspective urban morphology in momentary stress prediction. GPS embeddings emerge as the second most important modality, underscoring the significance of dynamic spatial exposure in stress prediction. This finding provides direct empirical support for the theoretical concerns raised by the UGCoP and NEAP,^10,11^ demonstrating that individuals’ real-time geographic locations and the environmental contexts they encounter along their trajectories are critical determinants of momentary stress that static residential-based assessments would fail to capture.

Beyond the multimodal fusion strategy, the design of a computationally efficient yet effective deep learning architecture tailored for moderate-scale spatiotemporal health data constitutes another contribution of this study. The architectural comparison identifies the center-aware MobileNet-style spatial adapter, group-wise MLP, and GRU temporal encoder as the optimal configuration. Specifically, the effectiveness of the center-aware mechanism provides empirical support for Tobler’s First Law of Geography in the context of stress modeling, confirming that nearby urban features exert a stronger influence on momentary stress than peripheral ones.^45^ The lightweight MobileNet-style adapter and GRU encoder, with their parsimonious parametrization, effectively balance model expressiveness and generalizability under limited sample sizes. The group-wise MLP respects the inherent heterogeneity among structured feature groups by learning dedicated nonlinear transformations for each semantic category before cross-group integration, capturing group-specific patterns that a unified processor would likely obscure.

The third contribution is the multilevel SHAP-based explainability framework, which provides comprehensive insights spanning temporal dynamics, spatial patterns, feature importance, and urban morphological attribution. While past study called for greater attention to the temporal dynamics of environment-stress relationships,^30^ most of the existing studies have largely treated environmental exposure as static contexts. We find instead that the influence of exposure is concentrated within roughly 25 min before the stress report and decays thereafter, and that different modalities act on different temporal scales. This gives a concrete basis for choosing the temporal resolution of exposure assessment in future EMA designs. Second, the relationship is strongly nonlinear. Rather than varying smoothly, several exposures show threshold effects, with stress rising sharply once noise exceeds about 55 dBA or PM_2.5_ exceeds about 25 μg/m^3^, and falling once visible greenery surpasses about 25%. The noise threshold is consistent with WHO community-noise guidance,^46^ while the PM_2.5_ threshold corresponds to the guideline value in the 2005 WHO air quality guidelines.^47^ The greenery pattern is also consistent with previous evidence on the restorative effects of urban green environments.^19,20^ The U-shaped patterns for income and education further reflect the socioeconomic complexity noted in the health literature.^9^ Third, the built environment matters through its morphology rather than its constituent parts. Greenery and open spaces are consistently associated with lower predicted stress, whereas the influence of roads and buildings depends on their surroundings, as major roads and dense high-rise clusters near intersections are associated with higher stress while quieter road segments and lower-density buildings embedded in greenery are not. This indicates that the same feature can carry opposite stress implications depending on its spatial configuration, so planning should target morphological context rather than individual features in isolation. Taken together, the short effective exposure window and the fine-grained spatial attributions point toward real-time, space-specific strategies for monitoring and relieving environmental stress, complementing the static, residence-based assessments behind most current policy.

This study has several limitations. First, the binary stress classification, while improving discriminability, sacrifices granularity in stress intensity. Second, the cross-sectional design within a single city limits generalizability. The specific thresholds we report should therefore be interpreted as context-specific findings for high-density Hong Kong rather than as universal values, and the transferable contribution of this study lies in the methodological framework, which provides a reproducible way of characterizing such nonlinear, time-lagged, spatially heterogeneous, and morphological patterns in other settings. Future studies should validate the framework and re-estimate these patterns across diverse urban contexts and populations. Third, the 30 min pre-EMA window, while capturing immediate exposure, may not fully account for cumulative or delayed stress effects. Although the 48-h duration survey captured daily patterns across both weekday and weekend contexts, it may have missed less frequent or nonroutine activities, exposure contexts, and their associated stress responses. Future research should explore longer temporal windows and continuous stress monitoring technologies to further advance our understanding of dynamic environment-stress interactions in urban settings. Moreover, the fixed, signal-contingent sampling captures momentary stress at predetermined times rather than emergent responses to discrete stressor events, which event-contingent designs could address in future work. Fourth, the study relied on self-reported momentary stress rather than physiological markers, and some reactivity inherent to repeated self-report cannot be fully excluded. Incorporating objective physiological indicators is an important direction for future research.

## Supplementary Material



## Notes

Text S1. Representation of four selected areas in Hong Kong; Text S2. Survey procedure and stress measures; Text S3. Data integration and cleaning; Text S4. Street-view panorama construction and synthesis, and POI taxonomy; Text S5. Model architecture and training; Text S6. Explainability analysis; Table S1. Demographic profiles of raw participants; Table S2. Demographic profiles of valid participants; Table S3. Predictive performance under different outcome granularities, comparing the binary outcome with finer three-class and six-class recodings of the original 1-to-6 stress responses; Table S4. Predictive performance under different minimum-records thresholds for retaining a sequence; Table S5. Predictive performance under three strategies for handling missing and extreme predictor values; Table S6. Selected 8-POI types and original subcategories; Figure S1. ROC curve comparison across model spatial adapters and temporal encoders; Figure S2. Confusion matrix comparison across model spatial adapters and temporal encoders; Figure S3. ROC curve of modality ablation; Figure S4. Confusion matrix of modality ablation (PDF)
