# Supplementary material for: An Explainable Multi-Modal Deep Learning Framework for Investigating the Influences of Dynamic Environmental Exposure on Momentary Stress
Source: Environ Sci Technol. 2026 Sep 21;60(39):27893–903. doi: 10.1021/acs.est.6c09466 (PMC13648242; doi:10.1021/acs.est.6c09466)
Supplement: Supplementary file 1 [file es6c09466_si_001.pdf]

Supporting Information for

An Explainable Multi-Modal Deep Learning Framework for Investigating the Influences of Dynamic Environmental Exposure on Momentary Stress

Linsen Wang[a], *Mei-Po Kwan*[a, b*], *Liuyi Song*[a]

[a] Institute of Space and Earth Information Science, Fok Ying Tung Remote Sensing Science Building, The Chinese University of Hong Kong, Shatin, Hong Kong, China.

[b] Department of Geography and Resource Management, The Chinese University of Hong Kong, Shatin, Hong Kong, China.

* Mei-Po Kwan (Corresponding Author)

Email: mpk654@gmail.com

This file includes:

Text S1 to S6

Tables S1 to S6

Figures S1 to S4

Text S1 Representing of four selected areas in Hong Kong

Participants are drawn from four strategically selected sub-districts: Kwai Tsing, Sha Tin, Central and Western, and Kwun Tong. Each district encompasses several Large Subunit Groups (LSUG), which function as Hong Kong's census tracts. These locations were deliberately chosen to reflect the city's remarkable urban heterogeneity. Hong Kong's developmental trajectory has shaped distinct neighborhood patterns across different eras[1]. Districts established before the 1960s (namely Central and Western along with Kwun Tong) display markedly different physical characteristics from their post-1980s counterparts, Sha Tin and Kwai Tsing. Earlier developments tend toward higher density construction, more complex street networks, and variable environmental conditions, while newer areas exhibit more planned layouts and infrastructure. The geographic distribution of these sites provides comprehensive territorial coverage, spanning all four of Hong Kong's major regions: Hong Kong Island, Kowloon, New Territories East, and New Territories West. This spatial arrangement captures the full breadth of the city's landscape variations. From an urban functional perspective, the selected areas represent diverse economic profiles and land uses. Central and Western merges affluent residential zones with a thriving commercial core. Sha Tin blends housing with retail amenities, attracting middle-income residents. Kwun Tong retains its industrial clusters with ongoing manufacturing activity. Kwai Tsing predominantly features residential complexes, sharing with Kwun Tong a relatively lower-income demographic. This multifaceted selection framework ensures our study sites encompass the full spectrum of built environment typologies, socioeconomic compositions, and environmental exposures (including variations in noise levels, green space accessibility, and air quality) that characterize Hong Kong's complex urban fabric.

Text S2 Survey procedure and stress measures

Recruitment and procedure. Participants were recruited through posters displayed around the selected communities and notices posted in local Facebook community groups. Individuals who expressed interest registered with the research team, and we verified that each registrant's place of residence fell within one of the four target areas. From this screened pool, participants were selected using a stratified sampling scheme informed by the demographic composition of each area in the Hong Kong population census (Table S1), so that the sample approximated the local population structure. Before participating, each selected participant attended a briefing session that included short training on the survey procedure and the equipment, and provided written informed consent. All monitoring devices were prepared and configured by the research team, and participants were only required to carry the device set during the survey period. At the end of the monitoring period, the devices were returned to the research team and the recorded data were checked for completeness. Upon completion, each participant received a subsidy of HKD 600.

Instruments. The noise meter (SLM-25) meets IEC 61672 Type 2 and ANSI S1.4 Type 2 standards

and records the A-weighted equivalent continuous sound level (LAeq, dBA). The ambient sensor (AirBeam; https://www.habitatmap.org/airbeam) records PM2.5 (μg/m³), temperature (°F), and humidity (%).

Momentary stress measure. The outcome was a single momentary item, "Do you feel stressed right now?", rated from 1 ("not at all") to 6 ("very severe"). We chose a single momentary item for two reasons. First, momentary stress, rather than trait or recalled stress, is the construct of theoretical interest in this study, and a momentary item captures the current state in context. Second, high-frequency EMA requires brief items to minimize respondent burden and the reactivity that longer or repeated multi-item instruments can introduce into the measured state, following established EMA methodology[2]. Although our specific item was not separately validated, single-item subjective stress measures of this form have demonstrated acceptable reliability and validity, including the single-item subjective level of stress (SLS-1) validated in Hong Kong community samples[3].

Activity-travel diary. Each evening, participants retrospectively recorded the activities and trips they had undertaken during the day, including the start and end time and the nature of each entry. Entries were classified into five categories: work/study, travel, personal affairs, leisure, and housework. To ensure data quality, each diary was cross-reviewed by two members of the research team, and entries with missing time segments were flagged and completed by the participant as soon as possible. These provided the activity-context variable that was temporally matched to each minute-level record.

Assessment completeness and quality control. Each participant could provide up to six EMA reports (three fixed prompts on each of two monitoring days), giving a maximum of 4,800 reports across the 800 participants. During fieldwork, the research team periodically reviewed diary and EMA completion, reminded participants to complete any missing diary entries, and, to preserve the integrity of the ecological momentary assessment (EMA), treated any prompt not answered within 30 minutes of its delivery as an invalid record. After applying this rule, 4,342 valid EMA reports were obtained, corresponding to a completeness of 90.5%. These quality-control procedures were applied uniformly across all participants and communities, which limits the scope for systematic, characteristic-dependent non-response.

Text S3 Data integration and cleaning

Resampling and aggregation. To integrate data from multiple sensors, all streams within the 30-minute window preceding each EMA record were resampled to a uniform one-minute resolution. PM2.5, temperature, and humidity were averaged within each minute. Because the A-weighted sound pressure level cannot be linearly aggregated, LAeq was computed as the one-minute equivalent continuous level following ISO 1999:1990[4]:

$$LAeq = 10\lg\left(\frac{1}{N}\sum_{t}^{t_n} 10^{0.1 LAeq_i}\right) \tag{1}$$

where N is the number of records within each minute, $LAeq_i$ is the raw LAeq at 30-second resolution, t is the start time of each minute, and t_n is the end time of each minute. GPS coordinates were aggregated to the per-minute median latitude and longitude, following[5].

Sequence construction. This procedure produced 4,342 candidate minute-level sequences of length 30, comprising 125,439 valid minute-level records. Sequences with fewer than 10 valid records within the 30-minute window were excluded (166 of 4,342 sequences, 3.8%), leaving 4,176 sequences for analysis. This threshold balances per-sequence completeness against sample retention; predictive performance was insensitive to its exact value, and a minimum of 10 gave the highest accuracy and ROC-AUC while retaining 96.2% of the candidate sequences (Table S4). For sequences shorter than 30 steps, following an approach of multi-modal fusion[6], a masking strategy was applied during training so that the absent steps did not contribute to the loss.

Missing and outlier handling. Within each retained sequence, two types of invalid records were addressed by spatiotemporal K-nearest-neighbor (ST-KNN) imputation[7]: device-missing records, and extreme values flagged by a two-sided 3×IQR threshold. The latter arise because low-cost mobile sensors are prone to transient errors that produce physically implausible spikes. We imputed these interior gaps rather than deleting them, because deleting time steps disrupts the temporal continuity on which the recurrent encoders rely, whereas ST-KNN imputation preserves it. Imputation also outperformed both deletion of extreme values and retention of the raw data (Table S5). To avoid relying on extensively reconstructed data, sequences with an excessive number of flagged or missing records, including those with fewer than 10 valid records, were discarded entirely, so imputation was applied only to sequences with minor gaps. Code to implement ST-KNN is available at the project GitHub repository (https://github.com/GEO-AI-for-health/Multi-Modal-Deep-Learning-Framework).

Text S4. Street-view panorama construction and synthesis, and POI taxonomy.

Panorama construction. For each location, four Google Maps images were captured at azimuths of 0°, 90°, 180°, and 270° (90° horizontal field of view, 0° pitch, 640 × 640 pixels each) and sequentially stitched into a composite panoramic view of 640 × 2,560 pixels.

Panorama synthesis for locations without coverage. For locations lacking SVI, panoramas were synthesized from the corresponding RSI using PanoGAN[8], originally trained on the Orlando-Pittsburgh dataset and fine-tuned on SVI collected from Hong Kong to adapt the model to the local

urban context. Synthesis quality was assessed using the Kullback-Leibler (KL) divergence score, which measures the divergence between the semantic-class distribution of the generated panoramas and that of the real panoramas under a pretrained classifier. A lower value indicates that the synthesized and real images are more similar, following the evaluation protocol[8]. Consistent with this literature, the score is reported as the mean and standard deviation across the validate set. The fine-tuned model achieved a KL score of 2.01 ± 0.13, compared with 3.11 ± 0.04 for the non-fine-tuned model, indicating that fine-tuning brought the synthesized panoramas closer to the real Hong Kong streetscape distribution. Code to download the RSI and SVI is available at the project GitHub repository (https://github.com/GEO-AI-for-health/Multi-Modal-Deep-Learning-Framework).

Points of interest. Based on Hong Kong's spatial functional classifications, the original Amap POI taxonomy was refined into eight categories: transportation, residence, industry, company, shopping, restaurant, entertainment, and recreation and tourism (Table S6).

Text S5. Model architecture and training.

Remote sensing encoder. RSI was encoded with the CLIP-RSICD-v2 vision encoder (https://huggingface.co/flax-community/clip-rsicd-v2), a fine-tuned version of OpenAI's CLIP model on remote sensing imagery[9]. The model uses a ViT-B/32 Vision Transformer image encoder fine-tuned on the remote sensing image caption dataset (RSICD). Because the ViT-B/32 encoder expects a 224 × 224-pixel input, each 1,024 × 1,024-pixel RSI was resized via the CLIPImageProcessor before being passed to the frozen encoder, producing a 50 × 768 embedding (one CLS token and 49 patch tokens on a 7 × 7 grid). These embeddings were compressed by a center-aware MobileNet-style spatial adapter[10]: a Gaussian-initialized spatial weighting prioritizes the geographic center of each tile (the participant's immediate surroundings), while a learned gating function retains peripheral information. The adapter produces an 80-dimensional RSI representation combining global semantic and spatially weighted local features.

Street-view encoder. SVI was segmented with Mask2Former using a Swin Transformer-Tiny backbone[11], which predicts a set of masks and labels through a masked-attention Transformer decoder trained on the Cityscapes dataset. Each 640 × 2,560-pixel composite panorama yielded pixel-level predictions across 19 Cityscapes categories, which were aggregated into category-wise area proportions and consolidated through domain-informed grouping into the eight SVI semantic indicators.

Structured-feature encoder. The SVI indicators, the mobile-sensor readings (noise, PM2.5, temperature, humidity), the POI composition and counts, the activity context, and personal SES were organized into five semantically coherent feature groups and processed through a group-wise MLP, yielding an 84-dimensional structured-feature embedding.

Spatial encoder. GPS coordinates were encoded using a multi-scale random Fourier feature encoder, which applies sinusoidal ([cos, sin]) transformations of the projected coordinates at four scales followed by an MLP, producing a 16-dimensional spatial embedding[12,13].

Temporal encoder and prediction. At each of the 30 one-minute steps, the RSI (80-D), structured (84-D), and GPS (16-D) embeddings were concatenated and processed by a two-layer GRU[14]. A masked mean pooling strategy handled variable-length sequences, and the aggregated context vector was passed to a linear classification head. The model was trained with a weighted focal loss (Eq. 4) and optimized using Adam with decoupled weight decay[15].

Ablation and evaluation. We compared spatial adapter types (MobileNet with center-aware weighting, MobileNet without center-aware weighting, CNN, multi-head attention, and direct pooling without adapter) and temporal encoders (GRU, LSTM, and MLP), and conducted a modality ablation removing one modality at a time from the best-performing architecture. We used 10-fold cross-validation, which provides a reliable, low-bias estimate of model performance[16], and which has been adopted in related environmental-exposure modeling work[5]. The data were randomly partitioned into 10 equally sized subsets and, in each fold, the model was trained on 9 subsets (90%) and validated on the remaining one (10%); performance was averaged over the 10 folds using accuracy, precision, recall, F1-score, and AUC-ROC.

Text S6. Explainability analysis.

SHAP attribution. Feature contributions were computed using a gradient-based SHAP method (the gradient explainer), which estimates expected gradients by averaging the gradient of the model output along paths between each input and a set of background reference samples drawn from the training data[17]. The number of background samples differed across the four analyses described below, reflecting their differing computational cost.

Feature-level analysis. SHAP values for the structured features were computed at the time step closest to the EMA report (T = 0), using 800 samples as both the background set and the set to be explained. Global importance was summarized by the mean absolute SHAP value across observations, and feature-outcome relationships were visualized with dependence plots fitted with a generalized additive model (GAM) to highlight nonlinear and threshold effects.

Modal-temporal analysis. The contribution of each modality at each of the 30 one-minute steps was computed using 50 background samples and displayed as a temporal heatmap, showing how the influence of each modality changes across the pre-EMA window.

Spatial analysis. The SHAP attribution associated with each GPS embedding was computed using 200 background samples and mapped to its geographic coordinates, producing a spatial distribution of stress-promoting and stress-suppressing locations.

Image-level analysis. For RSI, SHAP attributions were computed for the 49 patch tokens (7 × 7 grid) using 500 samples as both the background set and the set to be explained, then upsampled and overlaid on the original satellite image to indicate which parts of the surrounding urban form drove the prediction.

Table S1. Demographic profiles of raw participants

Variable		Overall	Sha Tin	Central and Western	Kwai Tsing	Kwun Tong
Age (Years)	18-30	299 (37.4%)	102 (32.6%)	34 (35.4%)	85 (40.5%)	78 (43.1%)
	31-45	311 (38.9%)	124 (39.6%)	39 (40.6%)	84 (40.0%)	64 (35.4%)
	46-68	190 (23.8%)	87 (27.8%)	23 (24.0%)	41 (19.5%)	39 (21.5%)
Gender	Female	548 (68.5%)	213 (68.1%)	64 (66.7%)	141 (67.1%)	130 (71.8%)
	Male	252 (31.5%)	100 (31.9%)	32 (33.3%)	69 (32.9%)	51 (28.2%)
Family Income (HKD/month)	Less 9999	52 (6.5%)	15 (4.8%)	6 (6.2%)	12 (5.7%)	19 (10.5%)
	10,000 -19,999	139 (17.4%)	66 (21.1%)	12 (12.5%)	30 (14.3%)	31 (17.1%)
	20,000 - 29,999	154 (19.2%)	65 (20.8%)	16 (16.7%)	41 (19.5%)	32 (17.7%)
	30,000 - 39,999	155 (19.4%)	55 (17.6%)	18 (18.8%)	42 (20.0%)	40 (22.1%)
	40,000 - 59,999	164 (20.5%)	63 (20.1%)	20 (20.8%)	44 (21.0%)	37 (20.4%)
	60,000 - 79,999	78 (9.8%)	27 (8.6%)	14 (14.6%)	23 (11.0%)	14 (7.7%)
	Over 80,000	58 (7.2%)	22 (7.0%)	10 (10.4%)	18 (8.6%)	8 (4.4%)
Education Level	Primary school or below	7 (0.9%)	2 (0.6%)	0 (0.0%)	1 (0.5%)	4 (2.2%)
	Secondary school	152 (19.0%)	66 (21.1%)	11 (11.5%)	33 (15.7%)	42 (23.2%)
	Post-secondary: Diploma / Certificate	85 (10.6%)	36 (11.5%)	8 (8.3%)	26 (12.4%)	15 (8.3%)
	Post-secondary: Sub-degree	24 (3.0%)	8 (2.6%)	1 (1.0%)	12 (5.7%)	3 (1.7%)
	Post-secondary: Degree	345 (43.1%)	110 (35.1%)	45 (46.9%)	100 (47.6%)	90 (49.7%)
	Master's degree or above	187 (23.4%)	91 (29.1%)	31 (32.3%)	38 (18.1%)	27 (14.9%)

Table S2. Demographic profiles of valid participants

Variable		Overall	Sha Tin	Central and Western	Kwai Tsing	Kwun Tong
Age (Years)	18-30	290 (38.3%)	99 (33.7%)	34 (37.8%)	83 (41.3%)	74 (42.8%)
	31-45	291 (38.4%)	114 (38.8%)	36 (40.0%)	80 (39.8%)	61 (35.3%)
	46-68	177 (23.4%)	81 (27.6%)	20 (22.2%)	38 (18.9%)	38 (22.0%)
Gender	Female	521 (68.7%)	200 (68.0%)	59 (65.6%)	136 (67.7%)	126 (72.8%)
	Male	237 (31.3%)	94 (32.0%)	31 (34.4%)	65 (32.3%)	47 (27.2%)

Family Income (HKD/month)	Less 9999	46 (6.1%)	12 (4.1%)	5 (5.6%)	10 (5.0%)	19 (11.0%)
	10,000 -19,999	131 (17.3%)	61 (20.7%)	12 (13.3%)	30 (14.9%)	28 (16.2%)
	20,000 - 29,999	152 (20.1%)	64 (21.8%)	16 (17.8%)	40 (19.9%)	32 (18.5%)
	30,000 - 39,999	146 (19.3%)	51 (17.3%)	15 (16.7%)	40 (19.9%)	40 (23.1%)
	40,000 - 59,999	152 (20.1%)	59 (20.1%)	19 (21.1%)	41 (20.4%)	33 (19.1%)
	60,000 - 79,999	76 (10.0%)	26 (8.8%)	13 (14.4%)	23 (11.4%)	14 (8.1%)
	Over 80,000	55 (7.3%)	21 (7.1%)	10 (11.1%)	17 (8.5%)	7 (4.0%)
Education Level	Primary school or below	7 (0.9%)	2 (0.7%)	0 (0.0%)	1 (0.5%)	4 (2.3%)
	Secondary school	148 (19.5%)	64 (21.8%)	10 (11.1%)	32 (15.9%)	42 (24.3%)
	Post-secondary: Diploma / Certificate	79 (10.4%)	32 (10.9%)	7 (7.8%)	25 (12.4%)	15 (8.7%)
	Post-secondary: Sub-degree	21 (2.8%)	6 (2.0%)	1 (1.1%)	11 (5.5%)	3 (1.7%)
	Post-secondary: Degree	329 (43.4%)	105 (35.7%)	44 (48.9%)	96 (47.8%)	84 (48.6%)
	Master's degree or above	174 (23.0%)	85 (28.9%)	28 (31.1%)	36 (17.9%)	25 (14.5%)

Table S3. Predictive performance under different outcome granularities, comparing the binary outcome with finer three-class and six-class recodings of the original 1-to-6 stress responses.

**Outcome granularity**	**Accuracy**	**Precision**	**Recall**	**F1**	**ROC-AUC**
Six-class	0.1848	0.1561	0.1820	0.1119	0.5062
Three-class	0.5616	0.5542	0.5540	0.5541	0.7040
Binary (main)	0.7826	0.8148	0.7904	0.8024	0.8277

Table S4. Predictive performance under different minimum-records thresholds for retaining a sequence.

**Minimum valid records**	**Sequences**	**Accuracy**	**Precision**	**Recall**	**F1**	**ROC-AUC**
0 (no exclusion)	4342	0.7604	0.8065	0.7622	0.7837	0.8217
10 (main)	4176	0.7826	0.8148	0.7904	0.8024	0.8277
20	4119	0.7785	0.7814	0.8563	0.8171	0.8266
30 (complete only)	3608	0.7439	0.8134	0.7415	0.7758	0.8265

Table S5. Predictive performance under three strategies for handling missing and extreme predictor value.

Strategies	Accuracy	Precision	Recall	F1	ROC-AUC
Raw (no removal, no imputation)	0.7659	0.787	0.7964	0.7917	0.8185
Remove extreme values, no imputation	0.746	0.7857	0.7639	0.7746	0.7654
ST-KNN imputation (main)	0.7826	0.8148	0.7904	0.8024	0.8277

Table S6. Selected 8-POI types and original subcategories

POI types	Subcategories
Transportation	Bus stops; car parks; metro stations; metro entrances; ports
Residence	Residential buildings; villas; dormitories
Industry	Factories; industrial buildings; industrial parks
Company	Company enterprises; office buildings
Shopping	Convenience stores; supermarkets; shopping malls; shopping streets; home and garden supplies; cultural & sporting stores; small commodities market; home appliance stores; department stores; flower, bird, fish and insect markets; other shopping
Restaurants	Chinese restaurants; Western cuisine; Japanese and Korean cuisine; Southeast Asian cuisine; vegetarian; other restaurants
Entertainment	Bars; amusement parks; theatres; resort; internet cafes; KTVs; chess and card rooms; cinemas; farmhouses; other entertainment
Recreation and Tourism	Urban parks; sightseeing spots; squares; religious places; aquariums; memorials; botanical gardens; zoos; other tourism; Fitness centers; sports complex; basketball; football; swimming; tennis; badminton; billiards; campsite; equestrian & horse racing; water sports; squash; golf; bowling; taekwondo; table tennis; ice sports; outdoor fitness area; rugby.

Figure S1. ROC Curve Comparison Across Model Spatial Adapters and Temporal Encoders

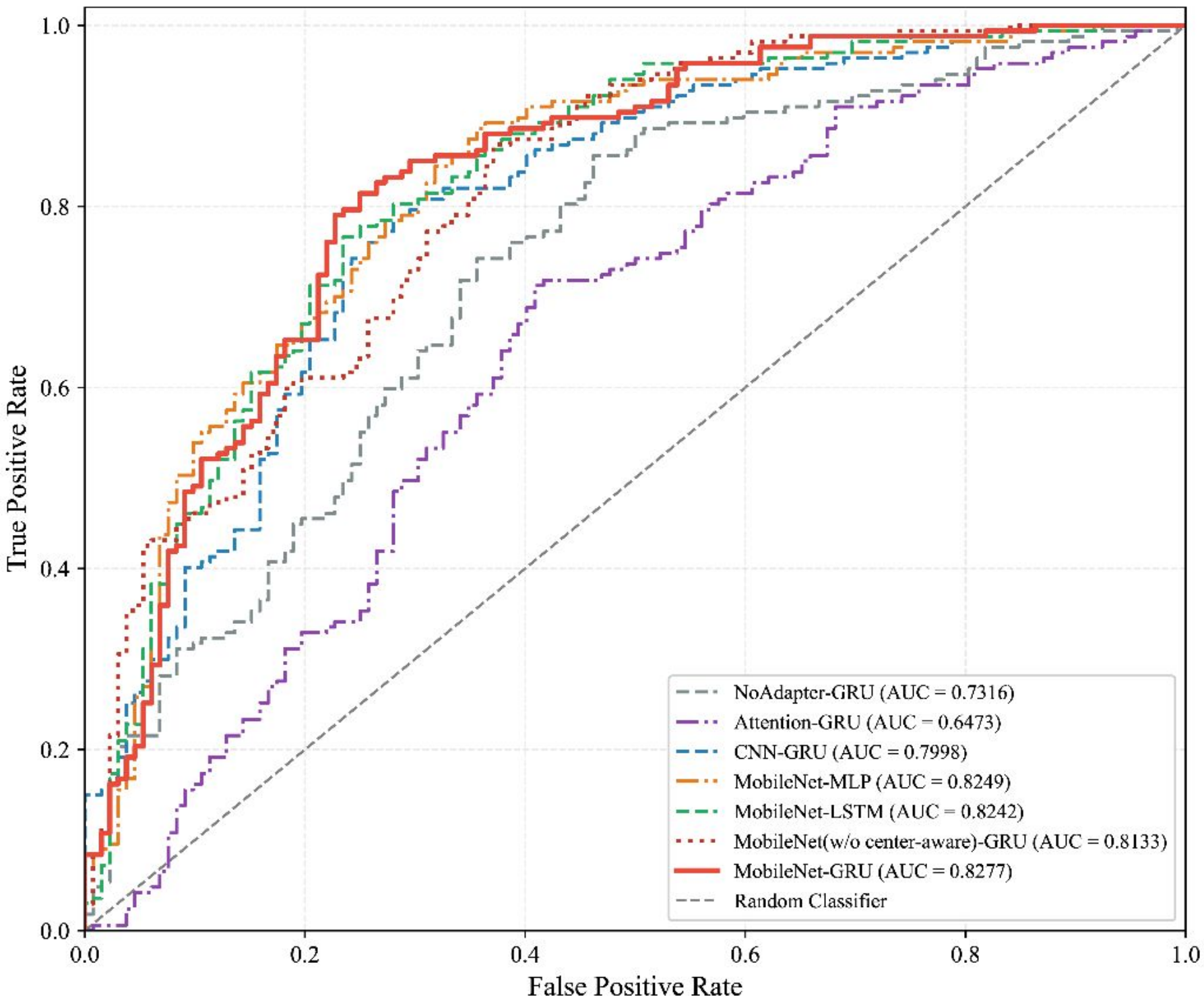

Figure S2. Confusion Matrix Comparison Across Model Spatial Adapters and Temporal Encoders

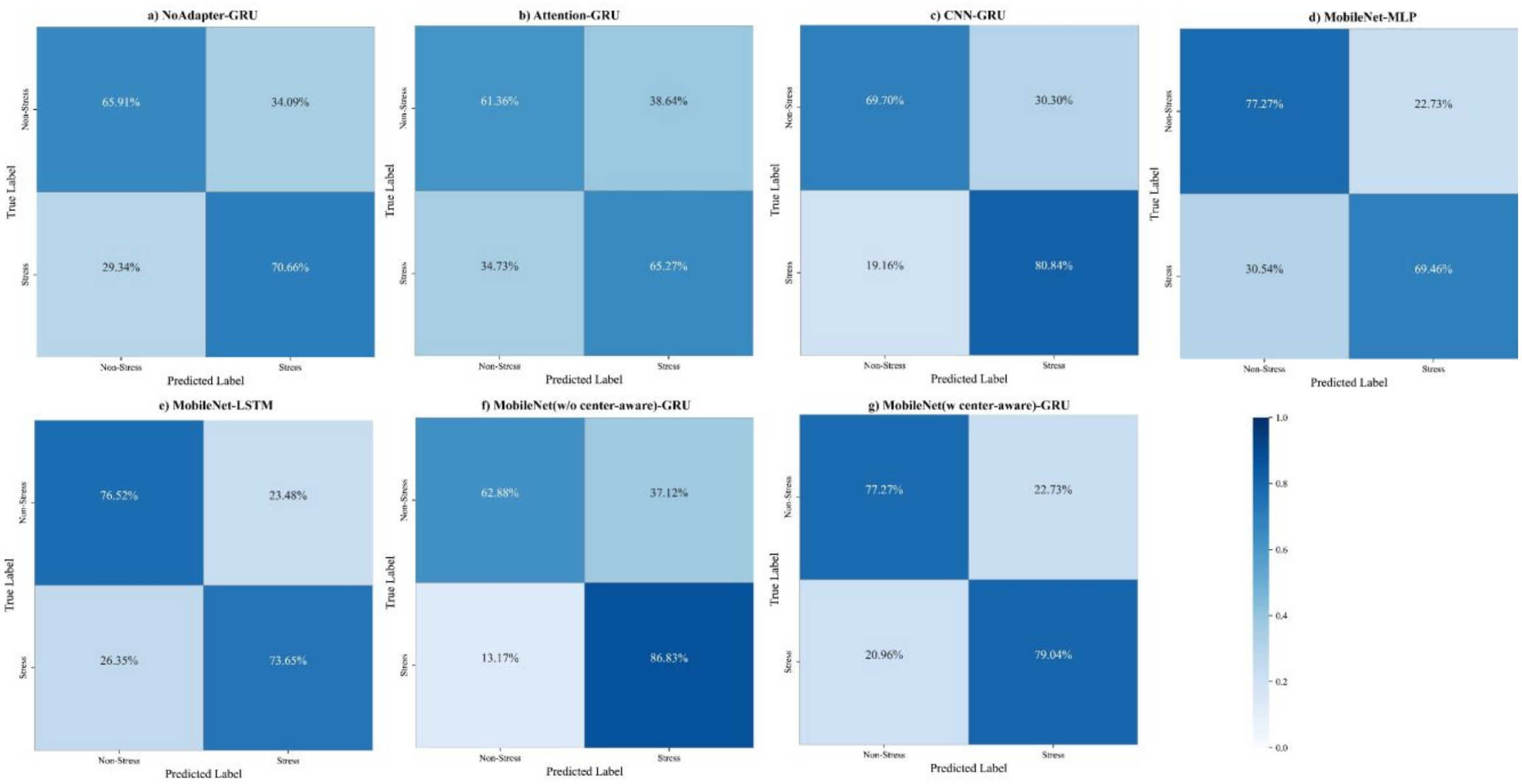

Figure S3. ROC Curve of Modality ablation

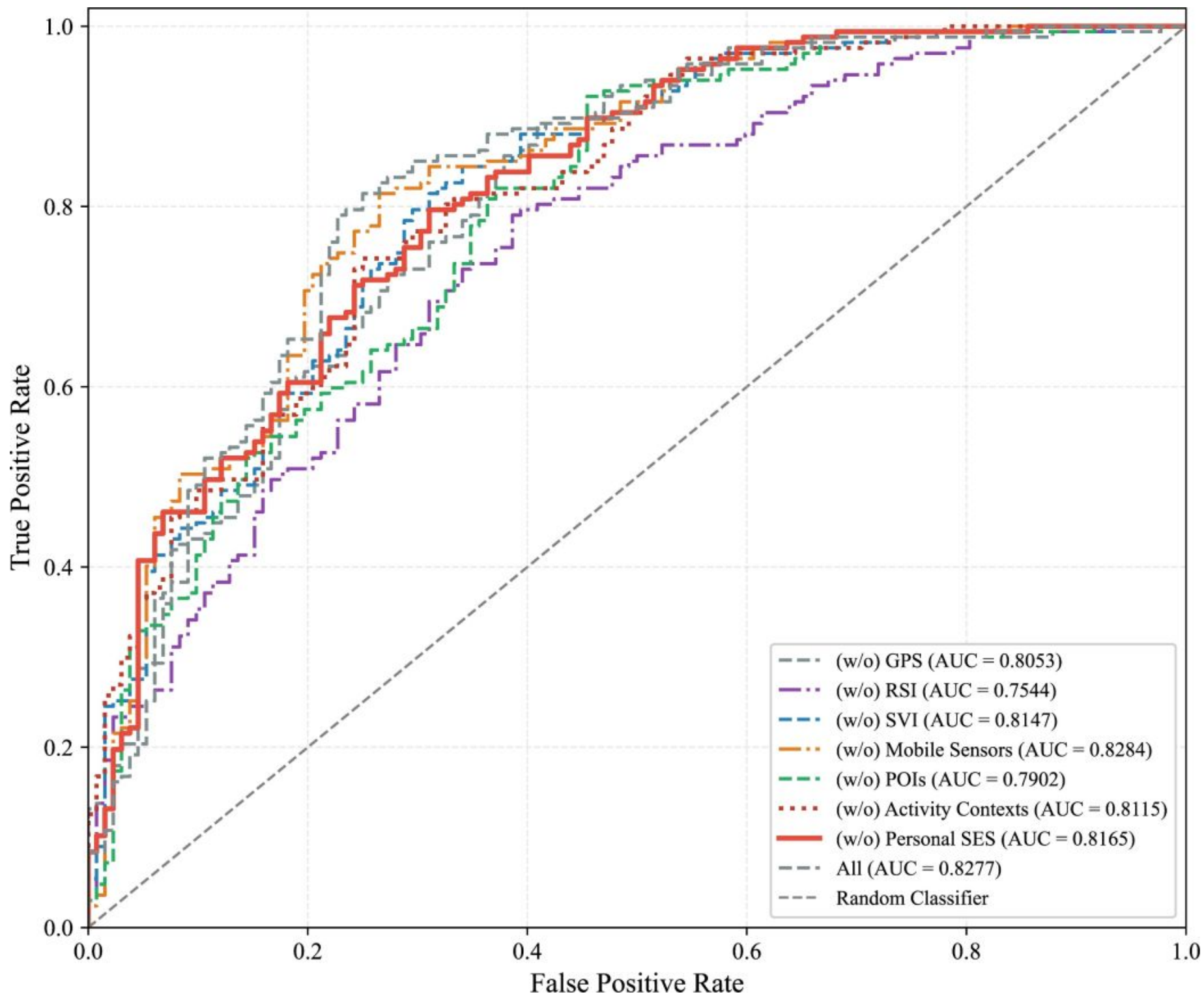

Figure S4. Confusion Matrix of Modality ablation

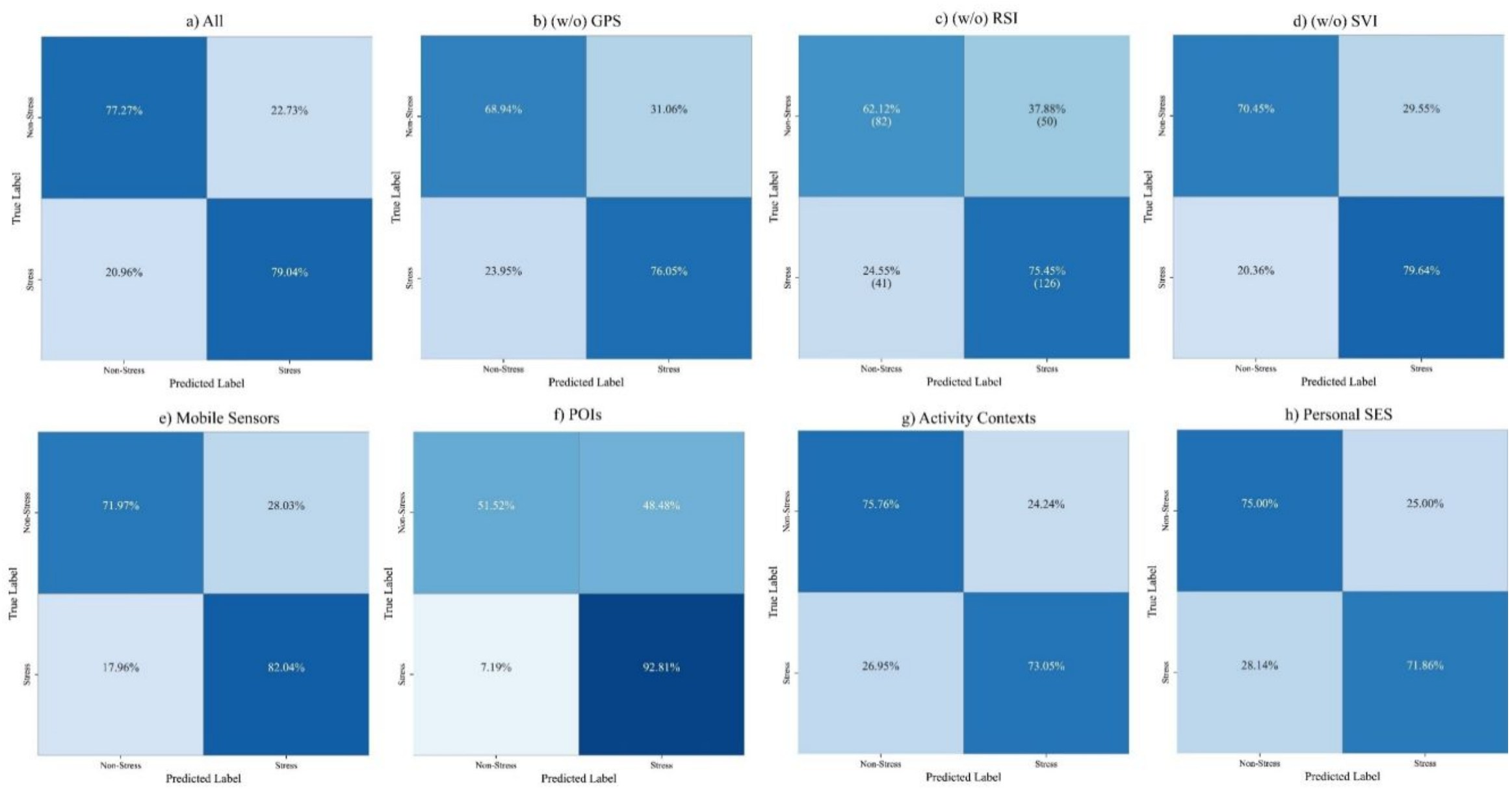

Reference

(1) Liu, Y.; Kwan, M.-P.; Wang, J. Analytically Articulating the Effect of Buffer Size on Urban Green Space Exposure Measures. Int. J. Geogr. Inf. Sci. **2025**, 39 (2), 255–276.

(2) Shiffman, S.; Stone, A. A.; Hufford, M. R. Ecological Momentary Assessment. Annu. Rev. Clin. Psychol. **2008**, 4, 1–32.

(3) Wong, S. M. Y.; Lam, B. Y. H.; Wong, C. S. M.; Lee, H. P. Y.; Wong, G. H.; Lui, S. S. Y.; et al. Measuring Subjective Stress among Young People in Hong Kong: Validation and Predictive Utility of the Single-Item Subjective Level of Stress (SLS-1) in Epidemiological and Longitudinal Community Samples. Epidemiol. Psychiatr. Sci. **2021**, 30, e61.

(4) International Organization for Standardization. Acoustics–Determination of Occupational Noise Exposure and Estimation of Noise-Induced Hearing Impairment; ISO 1999:1990; ISO: Geneva, Switzerland, **1990**. https://www.iso.org/standard/6759.html.

(5) Wang, L.; Kwan, M.-P. How Greenery Exposure Influences Noise Perception across Geographic Contexts. Landscape Urban Plann. **2025**, 263, 105444.

(6) Mühlematter, D. J.; Che, L.; Hong, Y.; Raubal, M.; Wiedemann, N. UrbanFusion: Stochastic Multimodal Fusion for Contrastive Learning of Robust Spatial Representations. arXiv Preprint, **2025**, arXiv:2510.13774.

(7) Cheng, S.; Lu, F.; Peng, P.; Wu, S. Short-Term Traffic Forecasting: An Adaptive ST-KNN Model That Considers Spatial Heterogeneity. Comput. Environ. Urban Syst. **2018**, 71, 186–198.

(8) Wu, S.; Tang, H.; Jing, X. Y.; Zhao, H.; Qian, J.; Sebe, N.; Yan, Y. Cross-View Panorama Image Synthesis. IEEE Trans. Multimedia **2022**, 25, 3546–3559.

(9) Radford, A.; Kim, J. W.; Hallacy, C.; Ramesh, A.; Goh, G.; Agarwal, S.; Sutskever, I. Learning Transferable Visual Models from Natural Language Supervision. In Proceedings of the 38th International Conference on Machine Learning; PMLR, **2021**; pp 8748–8763.

(10) Sandler, M.; Howard, A.; Zhu, M.; Zhmoginov, A.; Chen, L.-C. MobileNetV2: Inverted Residuals and Linear Bottlenecks. In Proceedings of the IEEE Conference on Computer Vision and Pattern Recognition; IEEE, **2018**; pp 4510–4520.

(11) Cheng, B.; Misra, I.; Schwing, A. G.; Kirillov, A.; Girdhar, R. Masked-Attention Mask Transformer for Universal Image Segmentation. In Proceedings of the IEEE/CVF Conference on Computer Vision and Pattern Recognition; IEEE, **2022**; pp 1290–1299.

(12) Tancik, M.; Srinivasan, P.; Mildenhall, B.; Fridovich-Keil, S.; Raghavan, N.; Singhal, U.; Ramamoorthi, R.; Barron, J. T.; Ng, R. Fourier Features Let Networks Learn High Frequency Functions in Low Dimensional Domains. Adv. Neural Inf. Process. Syst. **2020**, 33, 7537–7547.

(13) Liu, Z.; Lao, N.; Wang, Z.; Jiao, J.; Mai, G. GAIR: Location-Aware Self-Supervised Contrastive Pre-training with Geo-Aligned Implicit Representations. ISPRS J. Photogramm. Remote Sens. **2026**, 237, 166–182.

(14) Cho, K.; Van Merriënboer, B.; Gulçehre, Ç.; Bahdanau, D.; Bougares, F.; Schwenk, H.; Bengio, Y. Learning Phrase Representations Using RNN Encoder–Decoder for Statistical Machine Translation. In Proceedings of the 2014 Conference on Empirical Methods in Natural Language Processing (EMNLP); ACL, **2014**; pp 1724–1734.

(15) Loshchilov, I.; Hutter, F. Decoupled Weight Decay Regularization. arXiv Preprint, **2017**, arXiv:1711.05101.

(16) Kohavi, R. A Study of Cross-Validation and Bootstrap for Accuracy Estimation and Model Selection. In Proceedings of the 14th International Joint Conference on Artificial Intelligence (IJCAI); Morgan Kaufmann: San Francisco, CA, **1995**; Vol. 2, pp 1137–1143.

(17) Erion, G.; Janizek, J. D.; Sturmfels, P.; Lundberg, S. M.; Lee, S.-I. Improving Performance of Deep Learning Models with Axiomatic Attribution Priors and Expected Gradients. Nat. Mach. Intell. **2021**, 3 (7), 620–631.
